# Derepression of the *smvA* Efflux System Arises in Clinical Isolates of Proteus mirabilis and Reduces Susceptibility to Chlorhexidine and Other Biocides

**DOI:** 10.1128/AAC.01535-19

**Published:** 2019-11-21

**Authors:** H. Pelling, L. J. Bock, J. Nzakizwanayo, M. E. Wand, E. L. Denham, W. M. MacFarlane, J. M. Sutton, B. V. Jones

**Affiliations:** a Department of Biology and Biochemistry, University of Bath, Bath, United Kingdom; b School of Pharmacy and Biomolecular Sciences, University of Brighton, Brighton, United Kingdom; c National Infections Service, Public Health England, Salisbury, United Kingdom

**Keywords:** *Proteus mirabilis*, biocides, biofilms, catheter, chlorhexidine, efflux, lipopolysaccharide, urinary tract infection

## Abstract

Proteus mirabilis is a common pathogen of the catheterized urinary tract and often described as intrinsically resistant to the biocide chlorhexidine (CHD). Here, we demonstrate that derepression of the *smvA* efflux system has occurred in clinical isolates of P. mirabilis and reduces susceptibility to CHD and other cationic biocides. Compared with other isolates examined, P. mirabilis RS47 exhibited a significantly higher CHD MIC (≥512 μg/ml) and significantly greater expression of *smvA*. Comparison of the RS47 *smvA* and cognate *smvR* repressor with sequences from other isolates indicated that RS47 carries an inactivated *smvR*. Complementation of RS47 with a functional s*mvR* from isolate RS50a (which exhibited the lowest *smvA* expression and lowest CHD MIC) reduced *smvA* expression by ∼59-fold and markedly lowered the MIC of CHD and other cationic biocides. Although complementation of RS47 did not reduce MICs to concentrations observed in isolate RS50a, the significantly lower polymyxin B MIC of RS50a indicated that differences in lipopolysaccharide (LPS) structure are also a factor in P. mirabilis CHD susceptibility. To determine if exposure to CHD can select for mutations in *smvR*, clinical isolates with the lowest CHD MICs were adapted to grow at increasing concentrations of CHD up to 512 μg/ml. Analysis of the *smvR* in adapted populations indicated that mutations predicted to inactivate *smvR* occurred following CHD exposure in some isolates. Collectively, our data show that *smvA* derepression contributes to reduced biocide susceptibility in P. mirabilis, but differences in LPS structure between strains are also likely to be an important factor.

## Abstract (precis)

Proteus mirabilis is a common pathogen of the catheterized urinary tract and often described as intrinsically resistant to the biocide chlorhexidine (CHD). Here, we demonstrate that derepression of the *smvA* efflux system has occurred in clinical isolates of P. mirabilis and reduces susceptibility to CHD and other cationic biocides.

## INTRODUCTION

Biocides play an important role in infection control and are often used to remove microbes from equipment, surfaces, and skin prior to medical procedures (1–3). The rising incidence of multidrug resistance in nosocomial pathogens has further magnified the clinical value of biocides, and there is increasing reliance on these antimicrobials to reduce antibiotic usage through more effective infection control strategies (1, 4–6). However, despite their clinical significance, biocides are subject to far fewer regulations on their use than therapeutic agents, such as antibiotics (2). In many applications, there is also little or no direct evidence that biocide use is beneficial, and they are often used without a clear indication (3, 7, 8).

Commensurately, there is mounting concern that the inappropriate or overuse of biocides will lead to the emergence of biocide-resistant strains and could select for cross-resistance to antibiotics (1–5, 7, 9, 10). This would not only undermine the efficacy of these valuable agents but also exacerbate the problem of antimicrobial resistance and reduce our ability to control the spread of resistant organisms. This is exemplified by our previous work, which demonstrated that clinical isolates of Klebsiella pneumoniae can adapt to grow at increasing concentrations of chlorhexidine (i.e., become more resistant), and adaptation is associated with the acquisition of colistin resistance (4).

Chlorhexidine (CHD) is used extensively in health care settings both as a disinfectant and antiseptic. It is incorporated into a range of products, such as wipes, hand washes, wound dressings, irrigation solutions, and lubricating gels, and routinely applied to prevent infection (1, 2, 5, 7, 11–15). Examples of applications include the prevention of ventilator-associated pneumonia, the decontamination of skin at puncture or incision sites, control of wound infections, hard surface disinfection, decolonization of patients carrying opportunistic pathogens, such as methicillin-resistant Staphylococcus aureus (MRSA), and prevention of infections related to central venous catheterization (5, 7, 8, 13–16). CHD is also commonly used in a range of domestic health care products, such as mouthwashes and antiseptics (2, 7, 11, 14, 15), and as a teat wash in the veterinary field (17, 18). The diverse formulations and uses of CHD mean that in-use concentrations can vary from 0.015% to 0.02% for catheter maintenance solutions and wound irrigation products to 4% for surgical scrubs and other skin antiseptic products (4, 19). Therefore, pathogens present in the clinical environment are likely to encounter CHD at a wide range of concentrations, including sublethal levels that can potentially select for CHD tolerant strains.

Increased CHD resistance in adapted K. pneumoniae strains generated in our previous work was associated with mutations in a putative Tet repressor gene (*smvR*) that result in increased expression of the cognate *smvA* efflux system (4). The SmvA efflux pump is a member of the major facilitator superfamiliy (MFS) of transporters and was first described in Salmonella enterica serovar Typhimurium, where it was associated with efflux of quaternary ammonium compounds (QACs) and methyl-viologen resistance (20, 21). SmvA has now also been implicated in cationic biocide resistance in other members of the *Enterobacteriaceae* family and is potentially an important biocide resistance determinant in several clinically relevant Gram-negative species (2, 6). However, to date, we have only observed mutations in *smvR* that lead to reduced CHD susceptibility in K. pneumoniae and other species in laboratory experiments, and the clinical relevance of this biocide resistance mechanism remains unclear.

Proteus mirabilis is a prominent pathogen of the catheterized urinary tract and is associated with serious clinical complications (9, 22–24). This stems from the ability of P. mirabilis to form extensive crystalline biofilms on catheter surfaces, which result in obstruction of urine flow. In turn, this leads to the reflux of infected urine to the kidneys and precipitates the onset of pyelonephritis and septicemia (22–24). Resistance to CHD has been reported in P. mirabilis since the late 1960s, with initial studies conducted on isolates surviving the application of antiseptic preparations used in attempts to prevent catheter-associated urinary tract infections (25–27). The selection of strains resistant to high concentrations of CHD and a range of antibiotics, through repeated CHD exposure, has also been documented in clinical settings (9, 25–31). Nonetheless, many catheter maintenance solutions and lubricating gels still contain CHD, and it is likely that P. mirabilis is still often exposed to various concentrations of CHD as a nosocomial pathogen.

Despite its longstanding reputation as a CHD-resistant organism, the underlying mechanisms of CHD resistance in P. mirabilis are not well studied, and resistance is generally attributed to aspects of cell permeability. However, this species carries a homologue of the *smvAR* gene locus associated with adaptation to CHD in K. pneumoniae and other *Enterobacteriaceae* (4, 6). Therefore, we hypothesized that a greater understanding of CHD resistance in P. mirabilis could offer further insight into the development of clinically relevant mechanisms of CHD resistance, including the potential for *smvR* mutations to arise naturally in the clinical environment. Here, we identify and characterize a clinical isolate of P. mirabilis with defects in *smvA* regulation and high-level CHD resistance. We utilize this as a clinically relevant model organism to determine the role *smvA* overexpression plays in modulating susceptibility to a range of antimicrobials, as well as the impact on crystalline biofilm formation, which constitutes a key aspect of P. mirabilis pathogenicity.

## RESULTS

### Susceptibility of P. mirabilis clinical isolates to chlorhexidine.

To identify P. mirabilis clinical isolates with high-level CHD resistance, we determined the MIC of CHD for a panel of 10 isolates from urinary tract infections (Table 1). A wide variation in CHD MICs was observed for these isolates, with isolate RS50a exhibiting the lowest MIC. Conversely, isolate RS47 displayed considerably greater tolerance than all other isolates and was able to grow at the maximum CHD concentration that could be evaluated (Table 1).

**TABLE 1:** Susceptibility of P. mirabilis clinical isolates to chlorhexidine digluconate

Isolate	MIC (μg/ml)
B2	32–128
B4	32–64
RS1	16
RS6	32–64
RS17	16–64
RS18	32–64
RS28	128–256
RS40	32
RS47	≥512
RS50a	8–16

### Comparison of *smvAR* loci.

**FIG 1:**
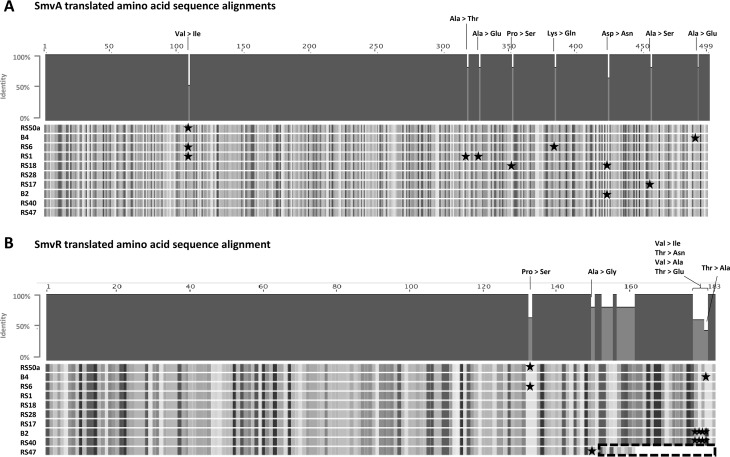
Comparison of SmvAR sequences. The *smvAR* locus was identified in draft genome sequences from P. mirabilis isolates analyzed for CHD susceptibility. Translated SmvR and SmvA amino acid sequences from all isolates were compared further by ClustalW alignment. (A) Shows alignments of SmvA sequences. B) Shows alignments of SmvR sequences. Amino acid residues that varied from the majority consensus between isolates are highlighted by symbols. The ∼22-amino acid truncation at the C terminus of RS47 SmvR is highlighted by the black dashed box.

To test the hypothesis that changes in *smvA* expression can modulate P. mirabilis CHD susceptibility, we first compared the *smvAR* genes from all 10 isolates (Fig. 1). Alignments of translated amino acid sequences from the SmvA efflux pump in these isolates showed a range of single amino acid substitutions primarily toward the C-terminal end (Fig. 1a). However, differences in SmvA sequences were not correlated with CHD susceptibility (Fig. 1a; Table 1). Conversely, alignments of the SmvR repressor sequence (which controls expression of *smvA*), revealed notable differences between the high-level CHD-resistant RS47 and other isolates (Fig. 1b). All differences between SmvR sequences were localized to a 30-amino acid (aa) region in the C terminus of the translated amino acid sequence and, in most cases, were related to single amino acid substitutions (e.g., RS50a, B4, and B2) (Fig. 1b). In contrast, RS47 exhibited a 22-aa truncation in the SmvR C terminus and substitutions in the 9 preceding amino acids (Fig. 1b). Further analysis of the RS47 *smvR* nucleotide sequence indicated a point mutation has resulted in the formation of a premature stop codon, which leads to truncation of the predicted amino acid translation.

### Impact of *smvR* mutations on *smvA* expression.

**FIG 2:**
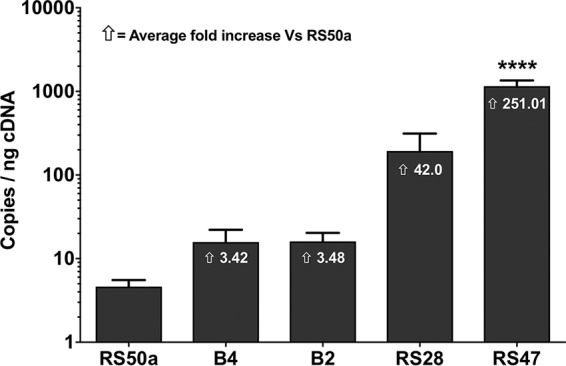
Expression of smvA in Proteus mirabilis strains. Quantitative real-time PCR was used to measure the expression of *smvA* in selected P. mirabilis isolates. Isolates chosen represent SmvR sequences with the range of amino acid variations observed (Fig. 2). A calibration curve of plasmid DNA containing an *smvA* fragment was used to determine absolute quantification. Expression of *smvA* is shown as copies per ng of cDNA template used. Data represent the mean of three biological replicates, with two technical replicates performed in each. Error bars show standard error of the mean. One-away ANOVA and Bonferroni’s *post hoc* test were performed comparing RS50a to other P. mirabilis strains. ****, *P* ≤ 0.0001 versus RS50a. The average fold difference in *smvA* expression of each isolate relative to RS50a is provided by numbers on bars.

To determine if the differences in SmvR amino acid sequences observed in these isolates corresponded to differences in expression of the cognate *smvA* efflux system, we measured expression of *smvA* in a subset of isolates using reverse transcriptase quantitative PCR (RT-qPCR) (Fig. 2). Isolates chosen represented the observed range of SmvR sequence types in amino acid alignments (Fig. 1). An overall correlation between *smvA* expression and susceptibility to CHD was observed (R^2^, 0.9; *P* ≤ 0.015). Isolate RS47, carrying a truncated SmvR and exhibiting the lowest susceptibility to CHD, displayed the highest level of *smvA* expression compared with the other 4 isolates tested (Fig. 2). Conversely, isolate RS50a, exhibiting the greatest susceptibility to CHD, displayed the lowest level of *smvA* expression. For isolates with intermediate CHD susceptibilities (B2, B4, and RS28), a greater but more variable level of *smvA* expression was observed, although these were not statistically significant differences compared with RS50a expression (Fig. 2).

### Expression of *smvA* following exposure to chlorhexidine.

**FIG 3:**
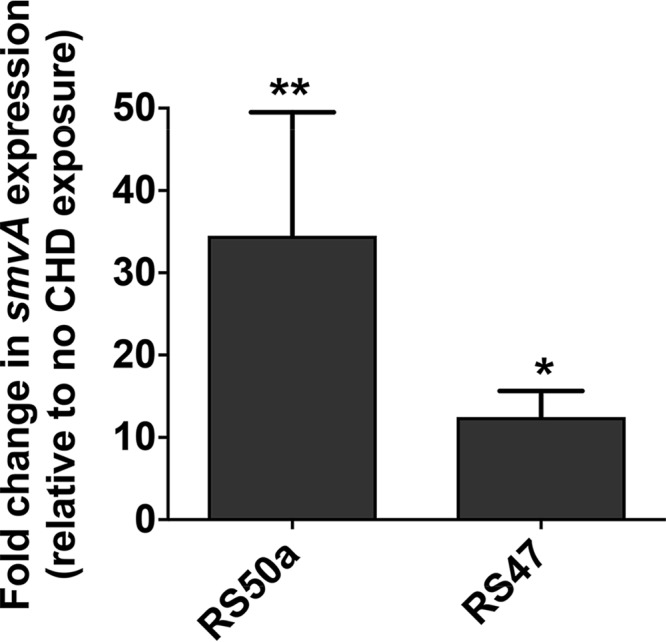
*SmvA* expression in Proteus mirabilis following chlorhexidine digluconate exposure. Quantitative real-time PCR was used to measure the expression of *smvA* in P. mirabilis following exposure to a sub-MIC of CHD. Exposure was to 4 μg/ml (0.25× RS50a MIC) for 15 min, and copies of *smvA* per nanogram cDNA template calculated. Data are expressed as fold change in *svmA* expression in CHD-exposed cells relative to unexposed cells. Data represent the mean of three biological replicates (with duplicated technical replicates for each), and error bars show standard error of the mean. Symbols indicate statistical significance of changes in *smvA* expression for each isolate compared with cells without CHD exposure. Statistical comparisons between CHD-exposed cells and unexposed cells of each isolate performed using a *t* test. *, *P* ≤ 0.05; **, *P* ≤ 0.01.

To determine if exposure to low levels of CHD influences the expression of *smvA*, we measured expression in RS50a (most CHD susceptible) and RS47 (least CHD susceptible) following exposure to sub-MIC levels of CHD (0.25× RS50a MIC). After CHD exposure, expression of *smvA* was significantly increased in both isolates compared with expression in unexposed subsamples of the same cultures (Fig. 3). However, RS50a displayed a notably larger relative increase in *smvA* expression following CHD exposure than RS47 (Fig. 3).

### Role of SmvA in modulating susceptibility to CHD and other antimicrobials.

**FIG 4:**
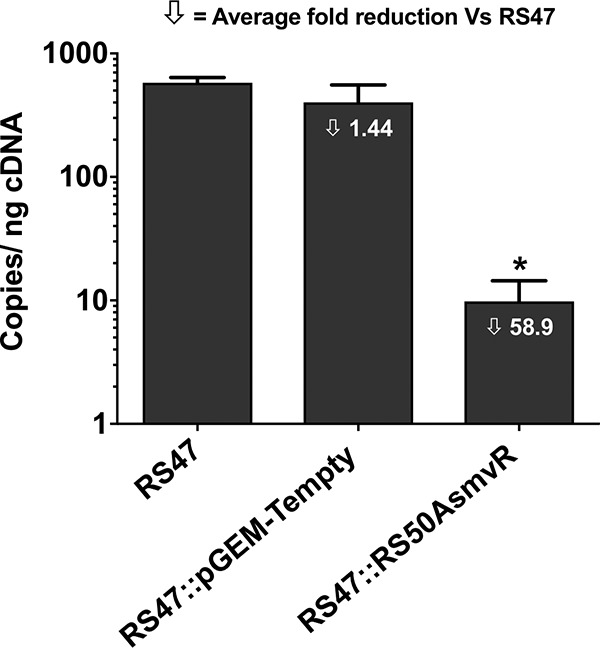
Impact of *smvR* complementation of RS47 *smvA* expression. Quantitative real-time PCR was used to measure the expression of *smvA* in the wild-type RS47 isolate and the derivatives RS47::RS50A*smvR* and RS47::pGEM-Tempty, harboring constructs with a functional copy of *smvR* or empty vector, respectively. Expression of *smvA* is shown as copies per nanogram of cDNA template from each strain. Data represent the mean of three biological replicates (with duplicated technical replicates performed in each), and error bars show standard error of the mean. One-way ANOVA and Bonferroni’s *post hoc* test were performed to identify significant differences in *smvA* expression between the wild type and transformants. *, *P* ≤ 0.01 versus RS47.

To confirm that overexpression of *smvA* was related to the CHD-resistant phenotype observed in RS47, a functional copy of *smvR* from isolate RS50a was introduced to RS47 on a plasmid vector. Measurement of *smvA* gene expression in the complemented RS47 derivative (designated RS47::RS50A*smvR*) confirmed that activity of the RS50a *smvR* in the RS47 background significantly reduced *smvA* expression compared with the wild type and RS47 carrying an empty vector (Fig. 4). MIC measurements confirmed that reduced *smvA* expression corresponded to an increase in CHD susceptibility (Table 2).

**TABLE 2:** MIC values of various antibiotics and biocides for wild-type and complemented isolates

Antimicrobial^*a*^	MIC (μg/ml) by isolate^*b*^			
	RS50a	RS47	RS47::pGEM-Tempty	RS47::RS50A*smvR*
Biocides				
CHD	8–16	≥512	>512	128–256
OCT	1–2	64–128	32–64	4–32
BZK	16	128	64–128	16–32
CET	8	256–512	256–512	32–64
CPC	4	256–512	128–256	16
CTAB	8	256–512	256–512	8
HDPCM	4–8	256	64–128	16–32
Antibiotics				
PMB	128 to 256	>2,048	>2,048	>2,048
NAL	4 to 8	4	2 to 4	4
FOF	128	256 to 512	256	256 to 512
GEN	16	8 to ≥16	4 to 16	4 to 16
CHL	32	16 to 32	8 to 16	8 to 16
TMP	4 to 8	4 to 8	1 to 4	2 to 4
CIP	0.016 to 0.06	0.06 to 0.25	0.03 to 0.125	0.03 to 0.06
AMX	4	2	*	*

a CHD, chlorhexidine digluconate; OCT, octenidine; BZK, benzalkonium chloride; CET, cetrimide; CPC, cetylpyridinium chloride; CTAB, cetrimonium bromide; HDPCM, hexadecylpyridinium chloride monohydrate; NAL, nalidixic acid; FOF, fosfomycin; GEN, gentamicin; CHL, chloramphenicol; TMP, trimethoprim; CIP, ciprofloxacin; AMX; amoxicillin.

b *, Isolates that required ampicillin selection were not tested against amoxicillin.

### Role of SmvA in modulating susceptibility to other antimicrobials.

To test the effect of altered *smvA* expression on susceptibility to other antimicrobial agents in P. mirabilis, we determined MICs of a range of antibiotics and biocides against the most CHD-susceptible isolate RS50a, the high-level CHD-resistant isolate RS47, and the complemented RS47::RS50A*smvR* (carrying a functional *smvR* from RS50a). For the antibiotics tested, no notable differences were seen between the strains, with the exception of polymyxin B (PMB), to which P. mirabilis is also considered to be intrinsically resistant. In this case, RS50a exhibited a notably reduced PMB MIC compared with RS47 and its derivatives (Table 2). All other clinical isolates displayed PMB MICs comparable to RS47.

Across all biocides tested, RS47 had considerably higher MICs than RS50a, ranging from ≥2- to 128-fold higher (Table 2). The presence of a functional copy of *smvR* in RS47::RS50A*smvR* resulted in increased susceptibility to most biocides, with MICs between 4-fold and 64-fold lower than the RS47 wild type (WT) (Table 2). This was most notable for the quaternary ammonium compounds cetrimide, cetylpyridinium chloride, hexadecylpyridinium chloride monohydrate, and cetrimonium bromide (Table 2). However, provision of a functional *smvR* did not reduce RS47::RS50A*smvR* MICs to values comparable to those of the RS50a donor strain in several cases. This was most apparent for CHD, where the RS47::RS50A*smvR* MIC remained 16-fold greater than that of RS50a (Table 2). MIC results for RS47 harboring the vector alone (RS47::pGEM-Tempty) were comparable to those of RS47 WT, with minor differences (≤2-fold) for some biocides not considered significant in these assays.

### Analysis of *smvR* mutations arising in chlorhexidine-adapted P. mirabilis populations.

In order to understand if CHD exposure selects for mutations in *smvR*, P. mirabilis isolates with the greatest susceptibility to CHD (RS50a, RS1, and B4) and lowest known *smvA* expression (RS50a and B4), were adapted to grow at increasing concentrations of CHD. Analysis of *smvR* in populations adapted to grow at 512-μg/ml CHD demonstrated a range of mutations in *smvR* had occurred in response to CHD exposure (Table 3). For CHD-adapted populations derived from isolates RS1 and B4, a variety of single nucleotide polymorphisms (SNPs) were detected, which were predicted to lead to either amino acid substitutions or the development of premature stop codons (Table 3). In the B4 CHD-adapted population, a duplication of 8 bp at position 282 occurred at a frequency of 7.4% and caused a frameshift mutation that ultimately leads to a premature stop codon at codon 108 (Table 3). Also identified in the adapted B4 population was a 14-bp deletion at position 467 which occurred at a lower frequency (1.3%) but also lead to a frameshift mutation, resulting in a premature stop codon at codon 157 (Table 3). In RS1 CHD-adapted populations, a SNP at position 10 occurred with a frequency of 13.7% and is predicted to lead to a premature stop codon at codon 4 (Table 3). In the RS50a population, a single SNP at position 70 was identified with 100% frequency, resulting in a glycine-to-arginine substitution at position 14 (Table 3).

**TABLE 3:** Analysis of *smvR* mutations arising in chlorhexidine-adapted populations

Parent	NT^*a*^ position	Mutation^*b*^	Frequency^*c*^ (%)	Amino acid change^*d*^
B4	7	C>T	1.5	R3C
	15	A>C	5.4	R5S
	85	A>G	10.0	T29A
	101	C>T	1.4	A34V
	282	(ATGATCAC)1>2	7.4	D95M, K96I, L97T, S98I, F99S, A100S, K101L, G102L, A103P, S104K, L105E, M106P, A108*
	307	G>T	2.2	A103S
	323	C>T	3.3	A108V
	460	C>T	1.4	R154W
	467	Δ14 bp	1.3	F156Y, G157*
	530	T>G	11.1	L177R
RS1	10	C>T	13.7	Q4*
	101	C>T	2.0	A34V
	292	T>C	15.3	S98P
	313	T>G	33.4	L105V
RS50a	70	G>A	100	G14R

a NT, nucleotide position in *smvR*.

b Mutations, NT substitution (e.g., C > T); duplication (e.g., [ATGATCAC] 1 > 2); deletion (e.g., Δ14 bp).

c Frequency, predicted frequency of sequences containing the mutation.

d Amino acid change, reference aa; aa position; new aa. Substitutions highlighted in bold indicate those where mutations lead to formation of a stop codon (*), predicted to truncate the translated protein at the indicated position.

### Role of *smvA* in crystalline biofilm formation and catheter blockage.

**FIG 5:**
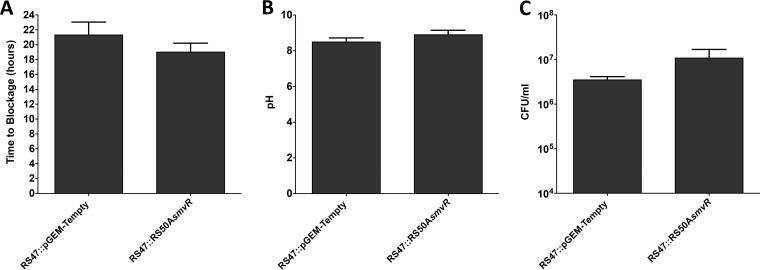
Impact of *smvA* overexpression on P. mirabilis crystalline biofilm formation. *In vitro* infection models simulating catheter-associated urinary tract infection were used to determine if *smvA* derepression influences crystalline biofilm formaiton. Models were used to simulate established infections with starting cell numbers of ∼10^8^ CFU/ml in residual “bladder” urine and ability to form crystalline biofilms assessed by time taken for catheters to become blocked. (A) Time taken for catheters to block. (B) pH of residual bladder urine at time of blockage. (C) Viable cells in residual bladder urine at catheter blockage. Data represent the mean from a minimum of four replicate experiments, and error bars show standard error of the mean. No statistically significant differences in time to blockage or pH and CFU/ml at blockage between isolates tested were identified (*t* test).

Efflux systems have recently been highlighted as important to the development of crystalline biofilms in P. mirabilis, which are a key aspect of P. mirabilis pathogenesis in the catheterized urinary tract (32, 33). This raises the potential for alterations in SmvA activity to also influence the formation of crystalline biofilms. To test this, we evaluated the ability of RS47 and its *smvR*-complemented derivative to form crystalline biofilms and block urinary catheters using an *in vitro* infection model (Fig. 5). However, no significant differences in ability to block catheters was observed between RS47::RS50A*smvR* and RS47::pGEM-Tempty (Fig. 5a). Both strains also showed comparable ability to elevate the pH of artificial urine and persist in these in models (Fig. 5b and c). Collectively, these results indicate that changes in *smvA* expression related to reduced biocide susceptibility do not influence the ability to form crystalline biofilms on catheters by P. mirabilis.

## DISCUSSION

Here, we demonstrate that mutations leading to the overexpression of the *smvA* efflux system are present in clinical isolates of P. mirabilis and reduce the susceptibility of this organism to a range of cationic biocides. Characterization of the clinical P. mirabilis isolate RS47, which exhibits high-level CHD tolerance, provided clear evidence that derepression of *smvA*, through inactivation of the cognate *smvR* repressor, is associated with increased tolerance to CHD and other cationic biocides. Subsequent experiments also indicated that *smvA* expression was increased upon CHD exposure, and *smvR* mutations arose in P. mirabilis populations after they were adapted to grow at increasing concentrations of CHD (up to 512 μg/ml). Conversely, *smvA* upregulation did not affect P. mirabilis susceptibility to the antibiotics tested in this study or modulate the ability to form crystalline biofilms on urethral catheters.

Defects in the *smvR* repressor analogous to those observed in RS47 were associated with the acquisition of CHD resistance in our previous studies of K. pneumoniae, following adaptation to increasing concentrations of CHD in the laboratory (4, 6). But P. mirabilis RS47 provides the first evidence that such mutations can arise in pathogens in the clinical environment. Other MFS transporters have also previously been linked with resistance to multiple biocides in both Gram-positive and Gram-negative species (2, 34–37). For example, MdfA of Escherichia coli and the QacA system from Staphylococcus aureus confer resistance to a wide range of cationic compounds from distinct chemical classes, including biocides (35–37). However, the results of this study further support our recent work highlighting SmvA as an important efflux pump for cationic biocides in multiple Gram-negative species and the potential for CHD exposure to select for mutations that promote cross-resistance to multiple biocides (6). Although not directly demonstrated in this study, we assume that overexpression of *smvA* leads to an increase in the quantity of SmvA efflux pumps present in the cytoplasmic membrane, which is responsible for the observed changes in phenotype. However, it should be noted that increased mRNA production does not always correlate with increased protein production, and other posttranscriptional or posttranslational mechanisms could influence the final amount of SmvA produced.

Nevertheless, while our data clearly indicate a contribution of the *smvA* system to reduced biocide susceptibility in P. mirabilis, it is more difficult to define where such changes in susceptibility would equate to clinically relevant biocide “resistance.” This stems from the use of biocides at a wide range of concentrations in numerous distinct products, the variety of applications in which biocides are used, and the inclusion of other compounds with potential antimicrobial activity in many products. Moreover, although actual in-use concentrations of biocides are often many times greater than MICs for bacterial pathogens, biocides are often required to work in challenging environments and parameters such as temperature, organic load, and exposure time all influence overall efficacy in terms of microbial killing. As such, definitions of resistance or clinical breakpoints are not well established for biocides, and interpretation of biocide resistance is often more complex than the simple relationship between the MIC of a bacterial strain and the in-use concentration of a biocide.

However, as P. mirabilis is a particularly problematic pathogen of the catheterized urinary tract associated with serious clinical complications, its potential to overcome the application of CHD-containing products used in catheter care is especially relevant (9, 26–31, 38). A notable example is bladder washout solutions containing 0.02% wt/vol chlorhexidine (200 μg/ml). At the time of writing, these products are available on the UK National Health Service (NHS) supply chain for urinary catheter maintenance and contain no other antimicrobial agents or ingredients expected to enhance biocide activity. Since the CHD MIC recorded for RS47 is considerably greater than the CHD concentration in this product, it can be reasonably proposed that *smvA* derepression could confer clinically relevant CHD resistance in at least some situations relating to bladder antisepsis. In addition, the propensity for P. mirabilis to form biofilms on catheter surfaces will contribute to further reductions in biocide susceptibility, and it is likely that cells embedded within biofilms will not be exposed to the full working concentration of antimicrobial agents contained within antiseptic products (9, 19, 32, 33, 39).

Notably, biocide susceptabilities modulated by *smvA* expression also included octenidine and a range of quaternary ammonium compounds (QACs), an observation that differs from previously published data from other *Enterobacteriaceae* (4, 6). Octenidine is increasingly used as a substitute for CHD and has been claimed to not promote resistance when used as recommeneded (40, 41). The overall contribution of *smvA* overexpression to octenidine susceptibility appeared to be much more pronouned than for CHD, with restoration of *smvR* activity reducing the octenidine MIC by 4- to 16-fold in RS47. However, the lowest concentration of octenidine currently contained in a commercially available product is 0.05% wt/vol for the Octenilin wound irrigation solution. As such, the RS47 MIC for octenidine remains well below in-use concentrations (provided the product is used as intended by the manufacturers), and it remains unclear if the reduced susceptibility we observed is relevant to clinical practice.

Nevertheless, our findings are in keeping with recent studies showing that P. aeruginosa and K. pneumoniae can adapt to octenidine challenge and develop stable reductions in susceptibility to in-use concentrations (6, 42). Furthermore, isolates of *Stapylococcus aureus* with increased octenidine MICs and minimal bactericial concentrations (MBCs) were found to rapidly emerge in clinical environments when octenidine use was introduced for decolonizing patients of MRSA (5). Our findings also fit well with data indicating that changes in *smvA* activity maybe a common mechanism of reduced susceptibility to both CHD and octenidine in a range of Gram-negative species (6). Overall, the phenotype of P. mirabilis RS47 suggests that reduced octenidine susceptibility in this species is already present in the clinical environment through *smvA* upregulation, and the implications for infection control warrant further monitoring.

Although our data clearly point to a role for *smvA* derepression in increasing P. mirabilis biocide tolerance, the examination of wider biocide and antibiotic susceptibilites in isolates we characterized also provides evidence that LPS structure is an important factor in the overall biocide susceptibility profile of P. mirabilis. In this regard, it is well established that properties of the Gram-negative bacterial cell envelope play a key role in the susceptibility of particular strains and species to biocides and other antimicrobials by inhibiting penetration of these agents into the cell and limiting their interaction with cellular targets (primarily the cytoplasmic membrane for cationic biocides such as CHD) (11, 12, 27, 43–45). Of particular interest in this context was the notably lower polymyxin B (PMB) MIC observed in RS50a than that in other isolates, which was not associated with *smvA* expression. PMB resistance in P. mirabilis stems from incorporation of the positively charged 4-amino-4-deoxy-l-arabinose (l-Ara4N) into the lipid A component of LPS (46–49). This modification reduces the overall negative charge of the outer membrane, leading to repulsion of cationic peptides, such as PMB, and a reduction in the susceptibility of P. mirabilis to these antimicrobials (45–49). Therefore, the lower PMB MIC observed in isolate RS50a is likely to be indicative of differences in LPS structure compared with other isolates, which increases the overall net negative surface charge of RS50a.

Because CHD is also a cationic compound, alterations in LPS net charge are also likely to modulate LPS permeability to CHD (1, 12, 44). If so, this feature of RS50a could also explain why restoration of *smvR* activity in RS47 did not reduce the CHD MIC to concentrations comparable to the RS50a *smvR* donor. Conversely, *smvR* complementation did reduce the RS47 CHD MIC to concentrations comparable to those found in other clinical isolates where *smvA* expression was analyzed (B2, B4, and RS28) and which also exhibited PMB MICs identical to RS47. Moreover, individual RS50a CHD-resistant mutants isolated from populations adapted to grow at 512 μg/ml CHD all exhibited PMB MICs comparable to RS47 and other isolates in this regard. It is also notable that previous CHD adaption experiments with K. pneumoniae resulted in activation of pathways controlling the incorporation of l-Ara4N into LPS and increased colistin resistance (4, 50).

Collectively, these data indicate that the overall susceptibility profile of P. mirabilis to cationic biocides is likely to result from the interplay between both LPS permeability and active efflux. While LPS structure is an important factor governing the initial entry of biocides, such as CHD, into the cell, mutations in *smvR* and overexpression of the *smvA* efflux system can confer additional protection and further reduce susceptibility. This hypothesis is also congruent with recent studies seeking to distinguish the specific relative contributions of active efflux and outer membrane diffusion to overall antimicrobial susceptibility in Gram-negative bacteria, which illustrate how these key barriers cooperate to confer protection against a wide range of structurally unrelated antibiotics (45). Although further study will be required to fully understand the relative contributions of LPS structure and active efflux to biocide susceptibility in Gram-negative species, the development of new biocides will likely benefit from more detailed and systematic studies of how existing compounds interact with both aspects of bacterial cells.

The contribution of SmvA to cationic biocide susceptibility in P. mirabilis and other species also raises questions regarding the exact mechanism by which efflux confers protection against these agents. MFS efflux systems, such as SmvA, are usually considered to be single component pumps localized to the inner membrane, where they work to remove toxic substances from inside the cell, or from the cytoplasmic membrane itself, into the periplasm (2, 51–53). However, the main lethal effect of cationic biocides, such as CHD and quaternary ammonium compounds (QACs), is considered to be disruption of cytoplasmic membrane structure and function (1, 2, 11). The action of biocides could also disrupt the function of MFS transporters through perturbation of the proton motive force on which they rely (2). Therefore, efflux systems that export substrates into the periplasmic space would not logically be expected to confer resistance to CHD unless there is also involvement with outer membrane transporters, such as OmpD and OmpW, which are linked to SmvA-mediated resistance to paraquat in *S*. Typhimurium (1, 2, 21, 54). One possibility is that SmvA does not work as a single component system but is instead part of a cell-envelope spanning complex with these proteins that is able to remove toxic compounds from the periplasm as well as the cytoplasm. Studies of MFS systems in other species have demonstrated that some MFS transporters do form tripartite systems with periplasmic linker proteins and outer membrane porins, analogous to the better characterized resistance-nodulation-division (RND) pumps, to transport substances out of the cell (51, 55, 56).

Consideration should also be given to the possibility that SmvA does not primarily modulate biocide susceptibility by exporting antimicrobials directly but instead through the export of substrates that contribute to wider physiological processes and stress responses (2). For example, single component pumps from the MFS, small multidrug-resistant (SMR), and multidrug and toxic compound extrusion (MATE) families are known to have roles in osmoregulation and expulsion of osmoprotectants from cells, which could conceivably contribute to offsetting membrane damage and reduced functionality caused by biocides, such as CHD (2). Further detailed research will be required to clearly elucidate the mechanism through which MFS transporters, such as SmvA, facilitate biocide resistance, and a better understanding of these systems is likely to improve knowledge of numerous physiological processes in bacterial cells.

In conclusion, this study provides further evidence that the SmvA efflux system is an important factor modulating susceptibility to cationic biocides among Gram-negative bacteria. Moreover, we provide the first evidence that mutations leading to overexpression of *smvA* and broad-spectrum increases in cationic biocide tolerance have already occurred in the clinical environment and elevate MICs to concentrations that are likely to undermine the clinical use of some antiseptic formulations. In this context, our work adds to existing evidence that CHD-containing catheter care products are of questionable clinical benefit and there is a well-established link between CHD usage and selection of multidrug-resistant strains of P. mirabilis (9, 26–31, 38). The continued availability of urinary catheter products containing CHD in the NHS supply chain is, therefore, cause for considerable concern. More broadly, the clinical importance of biocides in infection control alone should provide sufficient grounds for greater surveillance of biocide resistance and the impact this may have on clinical efficacy of antiseptics, other antimicrobials, and infection control practices. The possibility that SmvA may also work in synergy with changes to the outer membrane, which is linked with resistance to antibiotics of last resort, such as colistin, adds further weight to this argument. If so, then efforts to control the spread of antibiotic resistance could also benefit from a greater understanding of the mechanisms underlying biocide resistance.

## MATERIALS AND METHODS

### General culture and media.

Clinical isolates of Proteus mirabilis used in this study were obtained from The Royal Sussex County Hospital and Bristol Southmead Hospital. Table 4 provide details of isolates and derivatives used in this study. Unless stated otherwise, bacteria were cultured in lysogeny broth (LB) (10 g/liter tryptone, 5 g/liter yeast extract, and 10 g/liter sodium chloride) or tryptic soya broth (TSB) (17 g/liter pancreatic digest of casein, 3 g/liter enzymatic digest of soya bean, 5 g/liter sodium chloride, 2.5 g/liter dipotassium hydrogen phosphate, and 2.5 g/liter glucose) at 37°C with aeration. For growth on solid media for isolation of single colonies, LB agar without salt (1.5% wt/vol agar) or MacConkey number 3 agar was used.

**TABLE 4:** List of isolates and derivatives used in this study

Strain by bacterial species	Comment(s)	Source
P. mirabilis		
B2	Clinical isolate from urinary tract infection.	Royal Sussex County Hospital or Bristol Southmead Hospital
B4	Clinical isolate from urinary tract infection.	Royal Sussex County Hospital or Bristol Southmead Hospital
RS1	Clinical isolate from urinary tract infection.	Royal Sussex County Hospital or Bristol Southmead Hospital
RS6	Clinical isolate from urinary tract infection.	Royal Sussex County Hospital or Bristol Southmead Hospital
RS17	Clinical isolate from urinary tract infection.	Royal Sussex County Hospital or Bristol Southmead Hospital
RS18	Clinical isolate from urinary tract infection.	Royal Sussex County Hospital or Bristol Southmead Hospital
RS28	Clinical isolate from urinary tract infection.	Royal Sussex County Hospital or Bristol Southmead Hospital
RS40	Clinical isolate from urinary tract infection.	Royal Sussex County Hospital or Bristol Southmead Hospital
RS47	Displays high level chlorhexidine resistance (≥512 μg/ml) and de-repression of the smvA efflux system. Clinical isolate from urinary tract infection.	Royal Sussex County Hospital or Bristol Southmead Hospital
RS50a	Clinical isolate from urinary tract infection.	Royal Sussex County Hospital or Bristol Southmead Hospital
RS47:RS50AsmvR	Derivative of isolate RS47 harboring functional copy of *smvR* repressor of *smvA* cloned from RS50a. The relevant RS50a fragment encoding *smvR* and upstream region encoding native promoter is cloned into the pGEM-T vector, which confers resistance to ampicillin.	This study
RS47:pGEM-Tempty	Derivative of isolate RS47 harboring empty pGEM-T vector Used as control for studies of effect of *smvR* complementation in RS47:RS50AsmvR.	This study
E. coli		
JM109	Standard cloning strain. Used as intermediate host for plasmid constructs harboring RS50A *smvR* (used in complementation of RS47) or fragments of B4 *smvA* (used as qPCR standards).	Promega, UK

### Determination of MIC.

The MIC of antibiotics and biocides, including CHD (Sigma-Aldrich) was determined using a broth microdilution method in 96-well polypropylene plates (Greiner Bio-One) for cationic compounds (57) and 96-well polystyrene plates (Corning) for antibiotics. Wells containing TSB with doubling dilutions of antimicrobial were inoculated with ∼1 × 10^5^ CFU/ml log-phase P. mirabilis cells and incubated statically for 20 h at 37°C. Wells containing isolates which required ampicillin selection were supplemented with 100-μg/ml ampicillin. Growth was determined by measuring optical density at 600 nm (OD_600_). The MIC was defined as the lowest concentration that inhibited measurable growth.

### Genome sequencing and *smvAR* analysis.

To evaluate potential differences in the *smvAR* genes across P. mirabilis isolates, whole-genome sequences were obtained using the PHE Galaxy platform as previously described (6, 58). *smvAR* loci in P. mirabilis were identified based on homology to the K. pneumoniae *smvA* sequence (GenBank accession number SSJ85692). Translated amino acid sequences from individual *smvA* and *smvR* open reading frames (ORFs) in each isolate were recovered and aligned pairwise using Clustal W implemented in Geneious 9.8.1.

### Measurement of *smvA* expression.

Expression of *smvA* was measured by RT-qPCR using primers SMVA-F and SMVA-R (Table 5). RNA was extracted from mid-log-phase bacterial cells using the total RNA RNeasy PowerMicrobiome kit (Qiagen). Cells were harvested by centrifugation (13,000 × *g*), resuspended in 100 μl PBS and processed immediately. Extractions were conducted according the manufacturer’s instructions, including the optional addition of 100 μl phenol-chloroform-isoamyl alcohol at 25:24:1 (vol/vol/vol) saturated with 100 mM Tris (pH 8.0; Sigma-Aldrich) to the PowerBead tube prior to the addition of the sample. The recovered RNA was treated using the DNase Max kit (Qiagen) according to the manufacturer’s instructions. DNase-treated RNA was used as the template (500 ng per reaction) to generate cDNA using a QuantiTect reverse transcription (RT) kit (Qiagen). qPCR was carried out in 25-μl reactions using a Rotor-Gene Q cycler with a Rotor-Gene SYBR green PCR kit (Qiagen). Each 25-μl reaction contained 12.5 μl 2× Rotor-Gene SYBR green PCR master mix, 100-ng cDNA template, and 10 pmol of each forward and reverse primer made to volume with nuclease-free water. For each of three biological replicates measured, duplicate technical replicates were conducted. Negative controls consisted of reaction mixtures containing no template cDNA, and reaction mixtures containing DNase-treated RNA to confirm removal of contaminating chromosomal DNA. A calibration curve of DNA standards (pGEM-T easy carrying a fragment of *smvA* from P. mirabilis B4) (see also Table 4 and 5) was included in each replicate experiment to permit quantification of *smvA* transcripts.

**TABLE 5:** List of primers used in this study

Target	Primer	Sequence (5′–3′)	Product, application, comments
*smvA*	SMVA-F	TCGCCACCCTTATTGCCATT	qPCR primer for *smvA* expression
	SMVA-R	CGGCGACTAACTGTAAGCGT	qPCR primer for *smvA* expression
*smvA*	SMVA3-F	CCTCACTTTTCGGGACAACG	Amplification of *smvA* region encompassing target qPCR primers SMVA-F and -R. Used for construction of qPCR standard
	SMVA3-R	ACCTAAACGCGCTAGCCAAA	Amplification of *smvA* region encompassing target qPCR primers SMVA-F and -R. Used for construction of qPCR standard
*smvR*	SMVR-BAMHI-F	GGATCCCGTTGCAGGCATGCTCATAG	Amplification of RS50a *smvR* and upstream promoter region for complementation of RS47. Primers introduce flanking BamHI restriction sites (underlined bases).
	SMVR-BAMHI-R	GGATCCCGCCTCTGTGTATTCCGACT	Amplification of RS50a *smvR* and upstream promoter region for complementation of RS47. Primers introduce flanking BamHI restriction sites (underlined bases).
*smvR*	SMVR-2-F	GCGCGATTTAATCAGGTGGT	Primer for region internal to *smvR* to confirm expression by RT-PCR.
	SMVR-2-R	TTCTGGCGTTTGCAGTAACG	Primer for region internal to *smvR* to confirm expression by RT-PCR.
*smvR*	SMVR-FLANK2-F	CGTTGCAGGCATGCTCATAG	Amplification of *smvR* for BreSeq analysis in isolates B4, RS1, and RS50a.
	SMVR-FLANK2-R	CGCCTCTGTGTATTCCGACT	Amplification of *smvR* for BreSeq analysis in isolates B4, RS1, and RS50a.

### Measurement of *smvA* expression following exposure to chlorhexidine.

Overnight cultures were diluted to OD_600_ of 0.1 in 10 ml LB and incubated for 1.5 h with shaking at 37°C to prepare mid-log-phase cells. Two 1-ml aliquots of each culture were pelleted by centrifugation (13,000 × *g*) and resuspended in either LB or LB supplemented with 4 μg/ml CHD. The resulting cell suspensions were incubated at room temperature for 15 min, before being harvested by centrifugation (13,000 × *g*) and resuspended in 100 μl PBS and 500 μl RNAlater (Invitrogen). Samples were stored in RNAlater at room temperature overnight before processing. RNA extractions and RT-qPCR were conducted as described for measurement of *smvA* expression above, also using SMVA-F and SMVA-R primers (Table 5).

### Complementation of RS47 with a functional *smvR*.

The *smvR* from wild-type P. mirabilis isolate RS50a was amplified by PCR using primers SMVR-BAMHI-F and SMVR-BAMHI-R (Table 5) and Taq PCR core kit reagents (Qiagen). Resulting PCR products were purified using a QIAquick gel extraction kit (Qiagen) and ligated into the pGEM-T easy vector (Promega), according to the manufacturer’s instructions. Resulting constructs were introduced into chemically competent E. coli JM109 cells (Promega) according to the pGEM-T easy vector system transformation protocol, and transformants were selected on LB agar supplemented with 100 μg/ml ampicillin, 80 μg/ml 5-bromo-4-chloro-3-indolyl-β-d-galactopyranoside (X-Gal), and 0.5 mM isopropyl β-d-1-thiogalactopyranoside (IPTG). Selected colonies were screened for the presence of the desired construct by plasmid extraction (QIAprep Spin Miniprep kit; Qiagen) and restriction digestion with BamHI (Fast Digest BAMHI; Thermo Scientific). Expression of *smvR* from the native promoter was confirmed in the E. coli background by RT-PCR using primers SMVR-2-F and SMVR-2-R (Table 5). The resulting pGEM-T constructs expressing RS50a *smvR* (designated pGEM-T::RS50A*smvR*) or empty pGEM-T vector (pGEM-Tempty) were subsequently introduced to P. mirabilis RS47 by electroporation (0.1-cm gap cuvettes, 1.25 V, 25 μF, 200 Ω). Following electroporation, cells were recovered in super optimal broth (SOC) medium (20 mM glucose, 10 mM MgCl_2_, 10 mM MgSO_4_, 2.5 mM KCl, 10 mM NaCl, 20 g/liter tryptone, and 5 g/liter yeast extract) for 1 h at 37°C with shaking, before selection of transformants on nonswarming LB (NSLB) agar supplemented with 100 μg/ml ampicillin.

### Adaptation of P. mirabilis to chlorhexidine.

P. mirabilis isolates RS1, RS50a, and B4 were adapted to grow at elevated concentrations of CHD as described previously (19). Briefly, 50 μl of overnight culture was used to inoculate 3 ml of TSB supplemented with a starting concentration of 8 μg/ml CHD and incubated at 37°C. After 48 h of incubation, 50 μl of the initial culture was transferred to 3 ml of fresh TSB with double the previous concentration of CHD and incubation continued. This process was repeated for 14 days to a final concentration of 512 μg/ml CHD. Cells surviving in the final culture (512 μg/ml CHD) were passaged 10 times on tryptic soya agar (TSA) in the absence of CHD, and the whole culture was then stored at –80°C until required.

### Analysis of *smvR* mutations in CHD-adapted mutants using the BreSeq pipeline.

Total genomic DNA from each adapted culture was extracted using the Wizard genomic DNA purification kit (Promega). To amplify *smvR*, hi-fidelity DreamTaq green PCR master mix (Thermo Scientific) was used with primers SMVR-FLANK2-F and SMVR-FLANK2-R (Table 5) (10 pmol/primer) in 50-μl reactions with ∼10- to 20-ng DNA template. Resulting *smvR* amplicons were purified using a QIAquick PCR purification kit (Qiagen) and sequenced by Public Health England Genomic Services and Development Unit (PHE-GSDU) on an Illumina (HiSeq 2500) instrument, with a minimum of 150 Mb of Q30 quality data obtained for each culture. The *smvR* sequences obtained from each CHD-adapted culture were compared with the parental wild-type sequence using the Breseq pipeline (59). Breseq was run in polymorphism mode with a cutoff *P* value of ≤0.01 to identify and predict the frequency of sequences containing single nucleotide polymorphisms (SNPs), duplications, and deletions (59).

### *In vitro* models of the catheterized urinary tract.

Bladder models representative of the catheterized urinary tract originally described by Stickler et al. (60) were performed with minor modifications described by Nzakizwanayo et al. (33). Artificial urine (AU) was prepared as a concentrated 5× stock solution containing sodium disulfate (11.5 g/liter), magnesium chloride (hexahydrate) (3.25 g/liter), sodium chloride (23 g/liter), trisodium citrate (3.25 g/liter), sodium oxalate (0.1 g/liter), potassium dihydrogen orthophosphate (14 g/liter), potassium chloride (8 g/liter), ammonium chloride (5 g/liter), gelatin (25 g/liter), tryptic soya broth (5 g/liter), calcium chloride dihydrate (3.25 g/liter), and urea (125 g/liter). Stock solutions of urea and calcium chloride dihydrate were filter sterilized separately (0.45-μm nitrocellulose membrane; Sartorius, UK), and other components were sterilized by autoclaving. For in-use concentrations, all components were combined and diluted to 1× strength using sterile deionized water, with the final pH adjusted to 6.1.

The bladder, consisting of a double-walled glass chamber, was maintained at 37°C by a water jacket supplied from a circulating water bath. Size 14 all-silicone Foley catheters (Bard, UK) were inserted into the bladder and the retention balloons inflated with 10 ml sterile water. A drainage bag was attached to the catheter to form a sterile, closed drainage system. AU was supplied to the bladder at a constant rate of ∼0.72 ml/min. Models were inoculated with 10 ml of a bacterial culture containing ∼10^8^ CFU/ml which were allowed to establish within the bladder for 1 h before AU flow was activated. The number of viable cells in the bladder residual urine and the pH of the medium were measured at the start and at blockage.

### Data availability.

Genome sequences have been deposited in GenBank under the BioProject accession number PRJNA554808. Proteus mirabilis *smvA* and *smvR* sequences can also be found at GenBank under accession numbers MN265394 and MN265395, respectively.
